# Supplementary figures and images for: Seasonal and Spatial Dynamics of Fungal Diversity and Communities in the Intertidal Zones of Qingdao, China
Source: J Fungi (Basel). 2023 Oct 14;9(10):1015. doi: 10.3390/jof9101015 (PMC10607781; doi:10.3390/jof9101015)

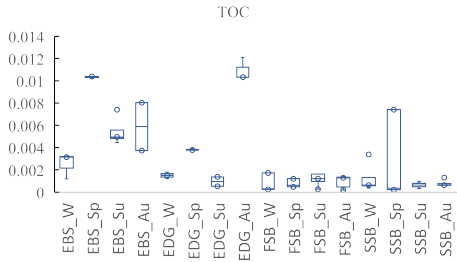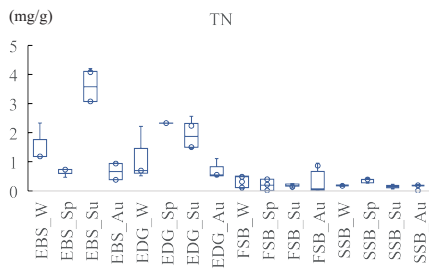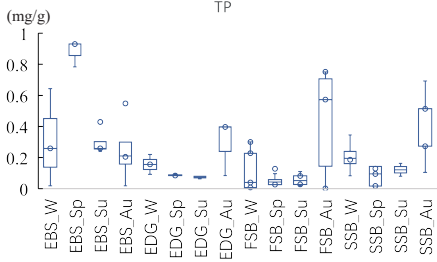

Supplement: Supplementary file 1 [file jof-09-01015-s001.zip › Figure S1.pdf]

Shannon index

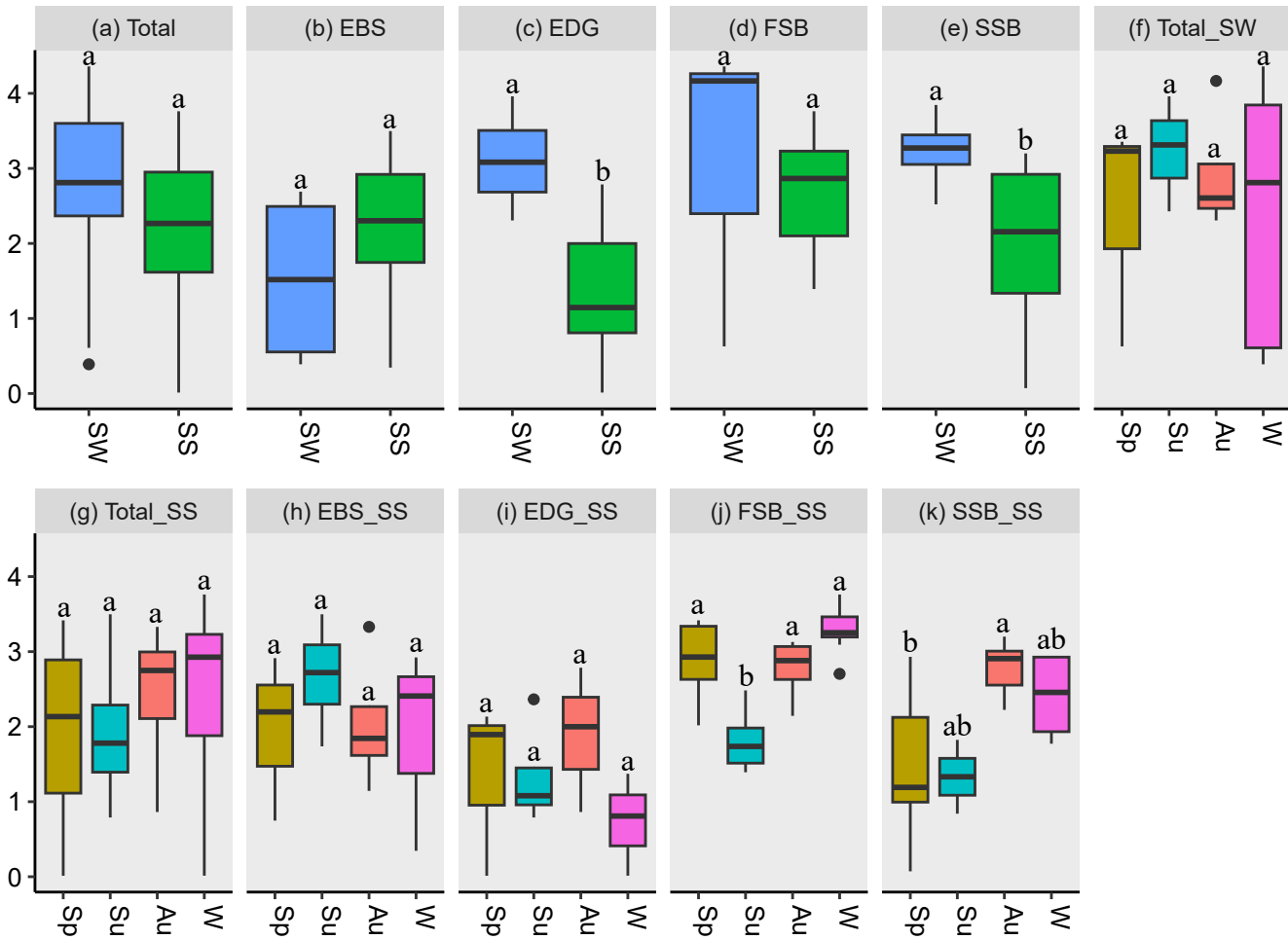

Supplement: Supplementary file 1 [file jof-09-01015-s001.zip › Figure S2.pdf]

Community similarity

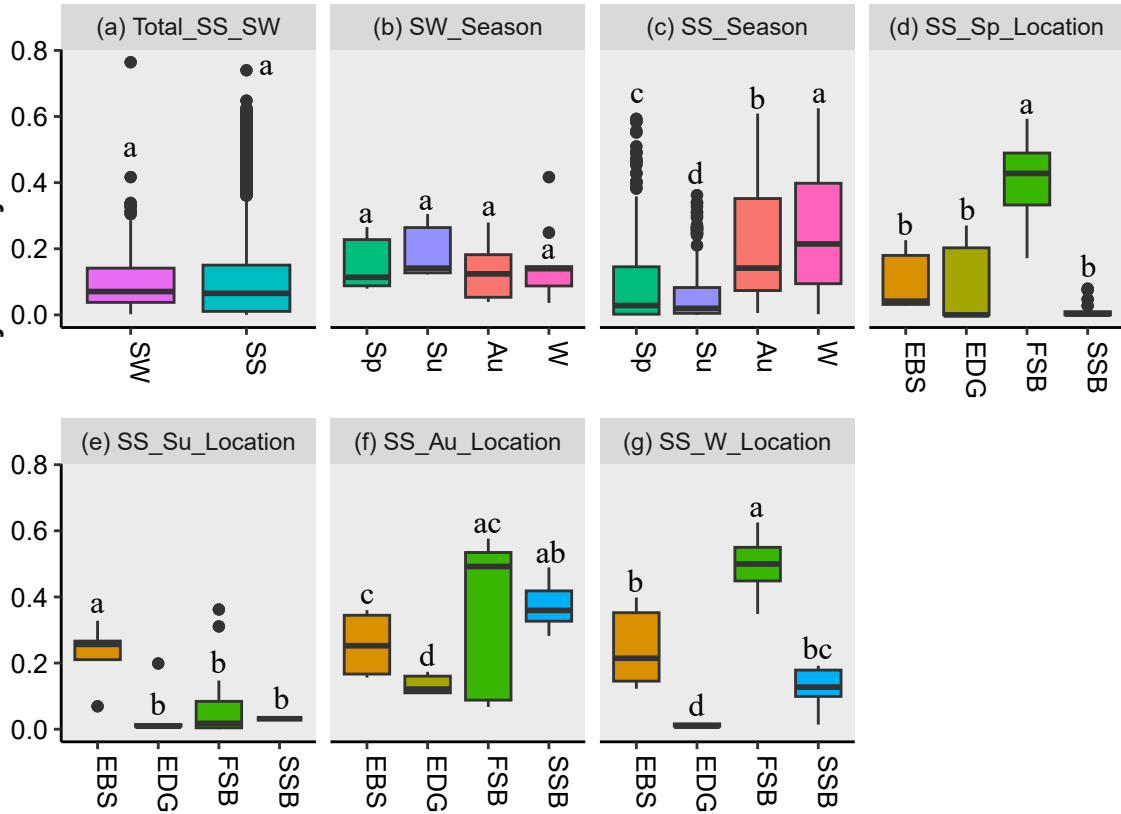

Supplement: Supplementary file 1 [file jof-09-01015-s001.zip › Figure S3.pdf]
